# Mothers’ experience of losing infants by death and its predictors in Ethiopia

**DOI:** 10.1371/journal.pone.0303358

**Published:** 2024-06-28

**Authors:** Addisalem Workie Demsash, Eyosiyas Yeshialem Asefa, Teshome Bekana

**Affiliations:** 1 Department of Health Informatics, Debre Berhan University, Asrat Woldeyes Health Science Campus, Debre Berhan, Ethiopia; 2 Midwifery Department, Debre Berhan University, Asrat Woldeyes Health Science Campus, Debre Berhan, Ethiopia; 3 Medical Laboratory Department, College of Health Science, Mattu University, Metu, Ethiopia; St. Paul’s Hospital Millennium Medical College, ETHIOPIA

## Abstract

**Background:**

Although infant deaths worldwide have reduced, many children die before their first birthday. Infant deaths are widespread in low-income countries, and information about the cause of death is limited. In Ethiopia, 53% of infants’ deaths occurred in their neonatal period, and 174 infants’ deaths occurred from 3684 births. Hence, this study aimed to assess mothers’ experiences with infant death and its predictors in Ethiopia.

**Methods:**

A total of 1730 weighted samples of mothers from the 2019 EDHS dataset, which was collected across the regions of Ethiopia, were included for analysis. A two-stage cluster sampling technique with a cross-sectional study design was used. All mothers whose children were under the age of 0–12 months were included in this study. Six count regression models were considered and compared using Akaike’s information criteria and Bayesian information criterion with STATA version 15 software. The strength of the association between the number of infant deaths and possible predictors was determined at a P-value less than 0.05, with a 95% confidence interval. The findings were interpreted by using the incident rate ratio.

**Results:**

A total of 46.3% of mothers had lost at least one infant by death in the last five years before the 2019 EDHS survey was held. The mean and variance of infant deaths were 2.55 and 5.58, respectively. The histogram was extremely picked at the beginning, indicating that a large number of mothers did not lose their infants by death, and that shows the data had positive skewness. Mothers under 25–29 years of age (IRR: 1.75, 95% CI:1.48, 2.24), and 30–34 years of age (IRR: 1.42, 95% CI: 1.12, 2.82), Somali (IRR: 1.47, 95% CI: 1.02, 3.57), Gambela (IRR: 1.33, 95% CI: 1.10, 2.61), and Harari (IRR: 1.39, 95% CI: 1.02, 2.63) regions, rural resident mothers (IRR: 1.68, 95% CI: 1.09, 1.91, and Protestant (IRR = 1.43, 95% CI: 1.14, 2.96), and Muslim (IRR = 1.59, 95% CI: 1.07, 2.62) religion fellow of mothers were associated with a high risk of infants’ deaths. Whereas, being rich IRR: 0.37, 95% CI: .27, .81) and adequate ANC visits (IRR: 0.28, 95% CI: .25, .83) were associated with a low risk of infant death.

**Conclusion:**

Many mothers have experienced infant deaths, and the majority of infants’ deaths occur after the first month of birth. Encouraging mothers to attend antenatal care visits, creating mothers’ awareness about childcare, and ensuring equal health services distribution and utilization to rural residents are essential to minimize infant death. Educating lower-aged reproductive mothers would be a necessary intervention to prevent and control infant deaths.

## Background

Infant mortality is the death of an infant before the first birthday, and the infant mortality rate (IMR) is the number of infant deaths per 1,000 live births [1]. It is a key marker of maternal and child health, and the overall health of societies as well [2]. IMR is useful for comparing the health status of a population over time or between populations at a single point in time. It permits comparisons of health systems and programs [3].

The world made remarkable progress in child death reduction in the past three decades, and millions of children had better survival rates in 2021 than in 2019 [4]. Globally, nearly 44% of all under-five deaths occurred during the neonatal period [5], 75% of deaths occurred within the first year of life, and an estimated 4.1 million infant deaths in 2017 [6]. Worldwide, the IMR has declined by 59%, from 93 deaths per 1000 live births in 1990 to 38 deaths per 1,000 live births in 2021 [4]. According to the Centers for Disease Control and Prevention, the IMR in the United States was 5.4 deaths per 1,000 live births in 2020 [7].

In low-income countries, the rate of child deaths is the highest [6, 8]. More than 98% of all child deaths occur in developing countries, where Sub-Saharan Africa takes half of those deaths [8]. Nearly 2.8 million and 1.5 million children’s deaths occurred in sub-Saharan Africa and Southern and Central Asia, respectively [9]. At least 1.2 million African children die in the first 28 days of life annually, and 850,000 of them do not live longer than a week after birth [10]. Low-income countries have made slow progress in reducing newborns deaths, especially deaths in the first week of life [11]. Child mortality in low-income countries is 20 times higher than in Austria and New Zealand [12]. According to the World Health Organization estimation, child mortality accounted for 150.3 billion US dollars in 2023 [13].

Almost half (49%) of the infants’ deaths occurred in Nigeria, India, Pakistan, the Democratic Republic of the Congo, and Ethiopia [14]. In Ethiopia, IMR is the highest in the world. Approximately, 12.2% of newborns die daily, and 37 neonates die per 1,000 live births [15, 16]. High infant mortality occurred in the Afar and Oromia regional states of Ethiopia [17]. Studies done in Eastern Ethiopia show that 53.0% and 47.0% of infants’ deaths occur in the neonatal and post-neonatal periods respectively [17]. Another study in the Tigray region of Ethiopia shows that 174 infants’ deaths occurred from a total of 3684 births [18]. According to the 2016 Ethiopian Demographic and Health Survey (EDHS) report, the IMR had a 50% reduction from 97 deaths per 1,000 live births in 2000 to 48 deaths per 1,000 live births in 2016. However, the reduction rate is insignificant [19], slow, and insufficient to meet the Sustainable Development Goal [20].

Infant deaths are linked to several causes and factors, which have led to a slow reduction of IMR and direct deaths in the last decade [21]. Malaria, acute respiratory infection, neonatal and post-neonatal pneumonia, diarrheal disease, birth asphyxia, and prematurity are the leading causes of infants’ deaths [17]. Moreover, maternal educational status and age [18], seasonal variation, children’s size and age [17], poor sanitation and unsafe drinking water, wealth status [14], distance, multiple numbers of children born [22], birth interval [23], and income [20] are significant contributing factors for infants’ death. Infant mortality is also significantly determined by home delivery, pregnancy, and birth complications, antennal care, breastfeeding, media exposure, and the consultation services received from health professionals [16].

Mothers’ enrolment in the health insurance system is associated with variation in access and utilization of health care services. Thus, mothers’ enrolment in the health insurance system could be a reason for mothers’ experience of losing an infant by death [24]. Delay or lack of vaccination [25], poor breastfeeding practice of mothers, poor awareness and knowledge of mothers, and poor hygiene could be factors for mothers’ experience about loss of infant by death [26].

Information on the cause of IMR is inadequate and inconsistent, and the available studies were mainly conducted in specific study areas that provide little information to understand the cause of infant deaths. Many of the previous studies have been done to assess the prevalence of infant mortality, studies regarding infant mortality over time are required. Infants’ death and their risk factors are very common in low-income countries like Ethiopia. The scarcity and inconsistent information about infants’ deaths would facilitate problems of child and maternal death.

The investigation regarding the cause of infant deaths at the population level is not well researched This study has been conducted based on nationally representative data that has the capability of providing accurate information about the number of infant deaths per month in Ethiopia. Understanding the prevalence of infant deaths and the contributing factors is critical for health policymakers and strategy developers looking to reduce infant mortality. Moreover, many previous studies have used 2016 EDHS data for statistical reporting about infants’ deaths, but many of the studies have not assessed the number of infant deaths over time using count regression models. Therefore, this study aimed to assess the number of mothers experienced with at least one infants’ death and associated factors using the Poisson count regression model to fill the existing knowledge gaps.

## Methods

### Study design and setting

The cross-sectional study design was conducted across the regions of Ethiopia. Ethiopia is located in the Horn of Africa and bordered by Eritrea to the north, Djibouti, and Somali to the east, Sudan and South Sudan to the west, and Kenya to the south. Ethiopia has nine regional states with two administrative cities. These are subdivided into different administrative units (68 zones, 817 Woredas, and 16253 Kebeles). Ethiopia has nearly a total of 129.7 million population with a 3.88 fertility rate [13]. Ethiopia is also a land of 15.5 million children under 5 years of age, nearly 8.4% of the total population [27].

### Data source

The 2019 Ethiopian Mini Demographic and Health Survey (EMDHS) dataset was used from the Demographic and Health Survey (DHS) program website (https://www.dhsprogram.com/).

The 2019 EMDHS data represents Ethiopia’s second DHS. The Ethiopian Federal Ministry of Health requested the Ethiopian Public Health Institute (EPHI) to implement the survey. The survey was conducted with the World Bank, UNICEF, and the United States Agency for International Development financial and technical support. The survey was conducted by EPHI in collaboration with the Central Statistical Agency (CSA) from March 21 to June 28, 2019. The 2019 EMDHS generates data for measuring the progress of the health sector goals set under the Growth and Transformation Plan, which is closely aligned with the Sustainable Development Goals [28].

### Sampling techniques and study population

The sampling frame used for the 2019 EMDHS is a frame of all census enumeration areas (EAs) created for the 2019 Ethiopia Population and Housing Census and conducted by the CSA. The census frame is a complete list of the 149,093 EAs, covering an average of 131 households, created for the 2019 EPHC. The sample for the 2019 EMDHS was designed to provide an estimation of key indicators for the country as a whole, for urban and rural areas separately, and for each of the nine regions and the two administrative cities.

Two-stage stratified cluster sampling was used. Each region was stratified into urban and rural areas. In the selected EAs, a household listing operation was done, and the results were used as a sampling frame for household selection in the second stage. Finally, a fixed number of households per cluster was selected. Samples of EAs were selected independently in each stratum through implicit stratification and equal proportional allocation.

**Fig 1:**
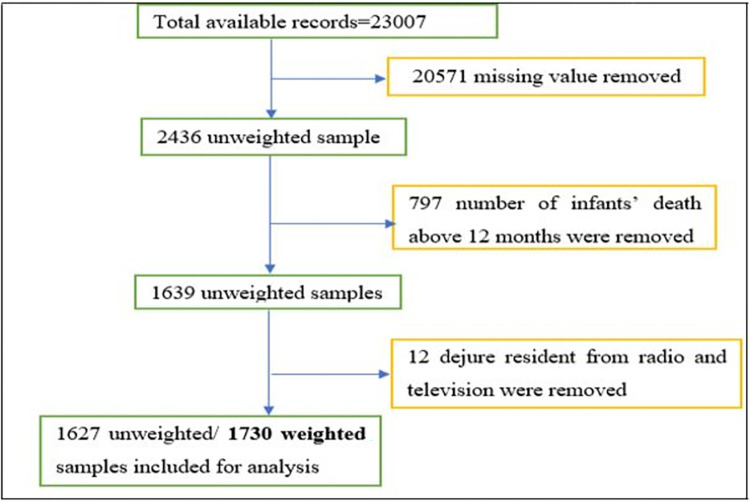
Sampling procedures of the study.

Standard questionnaires were adapted to reflect the population and health issues relevant to Ethiopia and donors. The household questionnaire was one of the questionnaires used to list all of the usual members of and visitors to the selected households. Basic information was collected from each listed person. In this study, all mothers whose children were under five years were the source population, and all sampled mothers whose children were under the age of 0–12 months were the study population [29]. The data on age and sex were used to identify eligible women for individual interviews. A total weighted sample of 1730 reproductive-age mothers who were interviewed about infant deaths in the preceding 5 years before the survey were included in this analysis [30]. The sampling procedure of this study is presented in **Fig 1**.

## Study variables

### Dependent variable

Number of infant deaths per mother.

### Independent variables

Socio-demographic characteristics of children such as region, place of residency, religion, child age, child sex, birth order, child twin, mothers’ age, mothers’ educational status, ANC visit, households’ wealth status, sex of household heads, and households’ media access were considered as possible independent variables.

### Operationalization

#### The number of infants’ deaths

Infants’ deaths are the deaths of infants before their first birthday [31]. Hence, the total numbers of mothers who lost their infants in the last five years of the 2016 EDHS interview were used to express the number of infants’ deaths.

#### ANC visits

The pregnant women had visited a health facility during their pregnancy for ANC services. Accordingly, the women had adequate ANC visits when they visited the health facility at least four times for ANC services, otherwise inadequate ANC visits [29, 32].

#### Media exposure

If the mothers had access to either radio or television or both, then the mothers had media exposure; otherwise the mothers had no media exposure [33, 34].

#### Religion

In this study, religion was used as an independent predictor for infants’ death. Studies show that various religions are associated with infant death [20, 35]. Thus, mothers’ religion was categorized as “Orthodox = 0”, “Catholic = 1”, “Protestant = 2”, “Muslim = 3”, and “traditional or other = 3”.

### Data processing and analysis

Data cleaning was performed to prepare the data for analysis and consistency assessment according to the objectives of the study. Variables were recoded to meet the desired classification. To ensure the representativeness of survey results at the national level [29, 36], sampling weights were applied during the analysis. The STATA version 15 software was used for data management and statistical analysis reports.

### Statistical models

In epidemiological studies and statistical methods of analysis, count regression analysis techniques are better for count response interests such as number of infants’ deaths [37]. Count regression models are suitable for the count-dependent variable, and the models are used to assess the prevalence or frequency of outcome interest over time [38]. These models can cope with the dependent variable’s non-normality, and the model does not require the dependent variable to be transformed or dichotomized. Since the dependent variable of this study are number of infant deaths per month, so count regression models are suitable for analyzing the number of infant deaths. Therefore, the Poison regression model (PRM), Hurdle Poisson (HP), Zero-Inflated Poisson (ZIP), Zero-Inflated Negative Binomial (ZINB), Negative Binomial (NB), and Hurdle Negative Binomial (HNB) of the count regression analysis models were considered for model comparison for this study. For the best-fit model, the strength of the association between the dependent and independent variables was determined at a P value of less than 0.05 with a 95% confidence interval (CI), and the incident rate ratio (IRR) was used to interpret the findings.

### Ethical approval and consent to participate

For this study, ethical approval was not necessary because the study was based on publicly available data sources from the measure DHS program website (https://www.dhsprogram.com/data/available-datasets.cfm). Informed consent from the study participants was also not applicable to the current study, and there are no attributes that uniquely identify individuals or households in this study.

## Result

### Socio-demographic characteristics

The majority (77.3%) of the mothers of infants were rural residents. Nearly four out of ten (41.5%), and one-fifth (20.9%) of mothers were from Oromia and Amhara regional states. 403 (23.3%) of mothers were between 40–44 years of age. The majority (72.0%) of mothers had no formal education. According to the wealth status of mothers, 754 (43.6%) of mothers were poor. The majority (81.4%) of household heads were male. 600 (34.7%) of mothers were orthodox religious flowers. Nearly three-fourths (75.4%) and nine out of ten (91.1%) of mothers had no radio and television, respectively (**Table 1**).

**Table 1:** Number of infant deaths per mothers’ sociodemographic characteristics in Ethiopia, 2019 EMDHS.

Variable	Category	Frequency (n)	Percent (%)
Place of residency	Urban	392	22.7
	Rural	1338	77.3
Region	Tigray	76	4.4
	Afar	20	1.1
	Amhara	361	20.9
	Oromia	718	41.5
	Somali	116	6.7
	Benishangul-Gumuz	25	1.4
	SNNPR	381	22.0
	Gambela	9	.5
	Harari	3	.2
	Addis Ababa	12	.7
	Dire Dawa	9	.5
Mothers’ age	15–19 years	27	1.6
	20–24 years	82	4.8
	25–29 years	269	15.5
	30–34 years	263	15.2
	35–39 years	321	18.5
	40–44 years	403	23.3
	45–49 years	365	21.1
Mothers’ educational status	No education	1245	72.0
	Primary	441	25.5
	Secondary	37	2.1
	Higher	7	.4
Sex of household head	Male	1409	81.4
	Female	321	18.6
Households’ wealth status	Poor	754	43.6
	Middle	382	22.1
	Rich	594	34.3
Religion	Orthodox	600	34.7
	Catholic	4	.2
	Protestant	584	33.8
	Muslim	520	30.0
	Traditional and other	21	1.3
Households have Television	No	1575	91.1
	Yes	155	8.9
Households have radio	No	1304	75.4
	Yes	426	24.6

### Models’ comparison and selection

The Log likelihood ratio (LLR), Akaike’s information criterion (AIC), and Bayesian information criterion (BIC) were used to compare various passion models, and the model with the smallest AIC, and BIC value was considered as a best-fit model [39].

**Fig 2:**
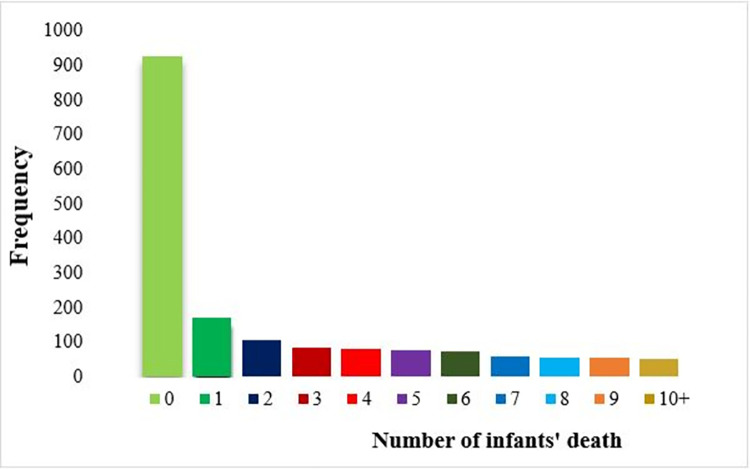
Histogram for the number of infants’ deaths per mother.

The mean and variance of infants’ deaths were 2.55 and 5.58 respectively (**Table 2**). So, the count Poisson regression model assumption is violated. This means the data is either over-dispersed or under-dispersed. Additionally, there is an excess number of mothers who did not lose their infants by death, and the variance was greater than the mean (**Fig 2**). The over-dispersion has been explained as heterogeneity that has not been accounted for an unobserved population that consists of several sub-populations. However, the sub-population membership is not observed in the sample. This excess variation may have caused incorrect inferences about parameter estimates, standard errors, tests, and confidence intervals. In the over-dispersed data, the mean is lower than the variance. So, the negative binomial, and zero-inflated Poisson (ZIP) regression model were the best to handle over dispersion that leads to much more variance, and excess zero. After calculating the AIC and BIC values of the comparable passion models, the AIC and BIC values of the ZIP model were 4774.044 and 4989.995 respectively, which was lower than other comparable models. Therefore, the ZIP model was the best-fit model to use to analyze the data (**Table 2**).

**Table 2:** Model comparisons.

Model selection criteria	Poisson regression	ZIP	ZINB	NB	HP	HNB
AIC	10796.81	**4774.044**	5832.09	6379.546	6298.01	7341.21
BIC	10980.37	**4989.995**	5912.33	6573.902	6573.35	7545.76
LLR	-5364.40	-2347.02	-3065.17	-3153.77	-3098.01	3274.46

### Prevalence of the number of infant deaths per mother

A total of 1730 weighed mothers whose children were under the age of 0–12 months were included in this study. A total of 803 (46.4%) mothers had lost at least one infant by death before their first birthday (12 months) in the last five years preceding the 2019 EDHS survey. From these, 2.9% of mothers had lost ≥10 infants by death, and 9.8% of mothers had lost one infant by death before their first birthday. The histogram was highly picked at the beginning. This means that there was an excessive number of mothers who had not lost an infant by death at the beginning (zero values). However, the number of infants’ deaths per mother declined less frequently. Plus, the number of infants’ deaths shows the variance (5.58) was greater than the mean (2.55). This indicates that the data had an over-dispersion (**Table 3 and Fig 2**).

**Table 3:** The number of infants’ deaths per mothers, 2019 EMDHS.

Number of infants’ death	Frequency	Percentage (%)
0	927	53.6
1	170	9.8
2	105	6.1
3	82	4.7
4	80	4.6
5	75	4.3
6	72	4.2
7	59	3.4
8	56	3.2
9	54	3.1
10+	50	2.9
Total	1730	
Skewness	1.36	
Kurtosis	0.52	

### Factors associated with the number of infants’ deaths in Ethiopia

In the zero-inflated Poisson regression model analysis, mothers’ age, region, place of residence, religion, rich wealth status, and adequate ANC visits were found as significantly significant factors in infants’ death in Ethiopia.

Mothers whose ages were under 25–29 and 30–34 years of age had 1.8 (**IRR: 1.75, 95% CI:1.48, 2.24**) and 1.4 (**IRR: 1.42, 95% CI: 1.12, 2.82**) times higher risk to lose their infant by death before their first birthday than 45–49 years of age mothers, respectively. Mothers from Somali, Gambela, and Harari regions had 1.5 (**IRR: 1.47, 95% CI: 1.02, 3.57**), 1.3 (**IRR: 1.33, 95% CI: 1.10, 2.61),** and 1.4 (**IRR: 1.39, 95% CI: 1.02, 2.63**) times more risk to lose infants by death before celebrating their first birthday than mothers lived in Tigray region, respectively.

Rural resident mothers were 1.7 (**IRR: 1.68, 95% CI: 1.09, 1.91**) times more likely to lose infants by death before their first birthday than urban resident mothers. Concerning the mother’s religion, Protestant, and Muslim religion fellow of mothers were 1.4 (**IRR = 1.43, 95% CI: 1.14, 2.96**), and 1.6 (**IRR = 1.59, 95% CI: 1.07, 2.62**) times more likely to lose infants by death before their first birthday than Orthodox region fellow of mothers, respectively. The rate of infants’ death for rich mothers declined by 40% (**IRR: 0.37, 95% CI: .27, .81**) as compared with poor mothers. The rate of infant death for mothers who had adequate ANC visits declined by 28% (**IRR: 0.28, 95% CI: .25, .83**) than mothers who had inadequate ANC visits (**Table 4**).

**Table 4:** ZIP regression fitted model for mothers’ experience with the number of infants’ deaths by possible potential predictors in Ethiopia, 2019 EMDHS.

Variables	Category	IRR	SE	Z value	P value	95% CI
Mothers age (ref:45–49 years)	15–19 years	1.01	.19	0.06	0.251	.70, 1.47
	20–24 years	1.03	.20	0.13	0.095	.69, 1.51
	25–29 years	**1.75**	**.16**	**-0.84**	**0.003** *	**1.48, 2.24**
	30–34 years	**1.42**	**.20**	**0.25**	**0.006** *	**1.12, 2.82**
	35–39 years	.98	.19	-0.08	0.075	.68, 1.43
	40–44 years	.89	.17	-0.63	0.126	.61, 1.29
Region (ref: Tigray)	Afar	1.05	.12	0.44	0.661	.84, 1.31
	Amhara	.88	.08	-1.37	0.171	.73, 1.06
	Oromia	1.12	.11	1.10	0.271	.92, 1.37
	Somali	**1.47**	**.14**	**2.13**	**0.014** *	**1.02, 3.57**
	Benishangul	.98	.11	-0.15	0.882	.80, 1.21
	SNNPR	1.14	.12	1.28	0.201	.93, 1.38
	Gambela	**1.33**	**.13**	**2.81**	**0.005** *	**1.10, 2.61**
	Harari	**1.39**	**.15**	**2.12**	**0.001** *	**1.02, 2.63**
	Addis Ababa	1.73	.29	3.28	0.071	1.25, 2.40
	Dire Dawa	1.07	.12	0.56	0.576	.85, 1.33
Residency (ref: Urban)	Rural	**1.68**	**.05**	**-0.26**	**0.007** *	**1.09, 1.91**
Mother’s educational Status (ref: no formal education)	Primary	**.90**	**.04**	**-2.45**	**0.014** *	**.83, .98**
	Secondary	.89	.09	-1.13	0.260	.73, 1.10
	Higher	.84	.13	-1.11	0.265	.61, 1.15
Religion (ref: Orthodox)	Catholic	.79	.13	-1.43	0.154	.57, 1.09
	Protestant	**1.43**	**.07**	**1.94**	**0.022** *	**1.14, 2.96**
	Muslim	**1.59**	**.07**	**0.80**	**0.009** *	**1.07, 2.62**
	Traditional and other	.94	.16	-0.39	0.695	.67, 1.30
Wealth status (ref: Poor)	Middle	1.05	.06	0.88	0.078	.95, 1.16
	Rich	**.37**	**.06**	**-0.47**	**0.015** *	**.27, .81**
Child twin (ref: Single birth)	1st of multiple	**.47**	**.10**	**-2.07**	**0.038** *	**.20, .98**
	2nd of multiple	.98	.07	-0.20	0.838	.86, 1.13
	3rd of multiple	1.37	.24	1.74	0.081	.96, 1.94
Sex of children (ref: Male)	Female	.92	.03	-2.55	0.061	.86, .98
ANC visits (ref: Inadequate)	Adequate visits	**.28**	**.07**	**-0.27**	**0.018** *	**.25, .83**
Birth order (ref: First order)	2–4 birth order	.98	.04	-0.41	0.682	.91, 1.06
	> = 5 birth order	.94	.04	-1.47	0.143	.86, 1.02
_cons		5.56	1.18	8.10	0.000	3.67, 8.43

*Significant at 95% CI, **IRR:** Incident rate ratio, **SE:** Standard error

## Discussion

In this study, 1730 weighted samples of mothers with children aged 0–12 months were included for statistical analysis. The study was conducted based on nationally representative data. Several count regression models that are suitable for analyzing integer response variables were tested. AIC and BIC were used as model comparison criteria. Six models were considered for model comparison. Accordingly, the zero-inflated Poisson regression model was selected as a best-fit model to assess the number of infant deaths per month in Ethiopia.

From a total of 1730 weighted samples of mothers, 46.4% had lost at least one child by death before celebrating their first birthday in the last five years before the 2019 EDHS survey was held. The current evidence was higher than similar studies conducted in Ethiopia [20], Bangladesh [40], and Nepal [41],. However, the finding was agreed with a study done in Uganda (27). This might be because mothers may not have received tetanus toxoid vaccination and adequate ANC service during pregnancy, may have had poor nutritional practices, and may not have received adequate essential healthcare consultation for infants’ care [40]. High IMR might be associated with long-term physiological effects on mothers [42], and the unmet need for family planning among reproductive married mothers that allow the presence of a short birth interval [40, 43]. Furthermore, high IMR may be associated with low institutional delivery, which increases the risk of death from birth asphyxia, poorly skilled assistance, mothers’ lack of awareness about the dangers of pregnancy, and mothers’ lack of preparation for birth [17].

Mothers under 25–29 years of age, and 30–34 years of age had1.8, and 1.4 times higher risks to lose their infants by death than mothers under 45–49 years of age, respectively. This finding is supported by studies done in Uganda [44], Ethiopia [17], and Malawi [45]. This could be because younger mothers have lower education and socioeconomic characteristics, which contribute to a higher IMR. Moreover, the mothers’ experience of child care increases as their age increases [46], and older mothers might have an experience of visiting health facilities for maternal and child healthcare services during pregnancy, institutional delivery, and child immunization, so they might have a better awareness of child care as compared to younger mothers [44]. Furthermore, young mothers are more likely to put off seeking appropriate medical care [47, 48], and infants born to young mothers are at a higher risk of respiratory infection, pneumonia, and birth asphyxia [49].

Children whose mothers were from Somalia, Gambela, and Harari were 1.5, 1.3, and 1.4 times more likely to lose infants by death than mothers who lived in the Tigray region, respectively. This is consistent with studies done in Ethiopia [17, 50] and Burkina Faso [51]. This might be due to variability in socio-economic conditions and the quality of healthcare services in administrative regions and weather conditions [17, 52]. Plus, several studies have reported that children from these regions faced poor vitamin A and iron supplementation, and inadequate resource distribution [29, 53, 54]. Moreover, mothers from pastoralist regions might have a negative attitude toward skilled birth attendants and a low perception of institutional delivery, which could be potential reasons for a reduction in maternal and child health service utilization and infant deaths [55–57].

Infants born to rural resident mothers were 1.7 times more likely to have a risk of death before celebrating their first birthday compared with infants born to urban resident mothers. This evidence is consistent with studies conducted in Ethiopia [20, 58], Kenya [59], and Nepal [60]. This might be because mothers who live in rural areas are less likely to have good infant care practices, poor access to and utilization of health services, and lower educational status [16]. Furthermore, rural households use shared toilet facilities, have unsafe drinking water, and children are vulnerable to various accidents [61]. Moreover, mothers from rural areas might have a low understanding of the importance of prenatal and postnatal care visits, and they have low media exposure [62, 63]. Therefore, children of rural resident mothers had a high risk of death before celebrating their first birthday.

Children born to protestant and Muslim religious fellow mothers had1.4, and 1.6 times higher risk of death before celebrating their first birthday than orthodox religious fellow mothers, respectively. This evidence is consistent with studies done in Ethiopia [20] and Nigeria [64]. This might be due to religious beliefs and norms preventing mothers from health facilities and receiving appropriate healthcare services such as contraceptives [65]. Additionally, the high cost might be invested or associated with a death anniversary, and Muslim husbands are religiously allowed to marry more than one wife. Thus, families might not be financially strong. After this, mothers might be restricted visit from visiting the health facility due to financial problems, and children might not access appropriate nutritional food. Moreover, husbands might oppose their wife to use maternal healthcare services [66].

The infants born from rich mothers had a 40% lower risk of death compared with those whose mothers were poor, respectively. This evidence agreed with studies done in Ethiopia [67, 68], Bangladesh [69, 70], Pakistan [71], and Nigeria [72]. This might be because children of the richest mothers have a lower chance of getting anemia than those of poor mothers; richest mothers are more likely to provide balanced and diversified food to their infants [73]; and they might have a better chance of accessing healthcare services than economically poor mothers [74, 75]. Moreover, children from lower economic statuses are vulnerable to various nutritional disorders, including anemia, stunting, malnutrition, and easily preventable diseases [76–78].

Infants whose mothers had adequate ANC were 28% less likely to die before their first birthday than mothers who had inadequate ANC visits. This evidence agreed with studies done in Ethiopia [79] and Nigeria [72]. This is because mothers who have adequate ANC visits might receive consultation from health professionals about how they care for and feed their children, resulting in good childcare practices [16]. During ANC visits, mothers might get a good awareness of pregnancy-related complications and pre-existing health conditions, and appropriate interventions are delivered for them [80]. Women and families receive behavioral change communication on personal hygiene, utilization of available services, and interventions [81]. Therefore, infants born from mothers who had adequate ANC visits have a low risk of death.

## Conclusion

The weighted sample of 1730 mothers were used to calculate the number of infant deaths in Ethiopia per month. Different Poisson regression models were tested. Excess zeros in the dataset were identified. As a result, the ZIP regression model provided the best-fit model for the data, and the majority of mothers did not experience infant deaths before a month. The mothers had lost their infants before their children were one month old. The age of mothers, regions, rural residency of the mothers, and religious status of mothers are significantly associated with infant mortality, but wealth status and adequate ANC visits of mothers are statistically associated with a reduction in infant deaths. Therefore, encouraging mothers to have more antenatal care visits, creating awareness for mothers regarding infant care, robust interventions in rural communities to minimize risk factors of infant death, and ensuring equity of health service distribution and utilization could be important solutions for infants’ deaths. Moreover, more efforts are required to educate reproductive mothers and enhance their wealth status to decrease infant mortality, and researchers also have recommended conducting more advanced studies such as causal inferential studies.

### Strengths and limitations of the study

This study was based on a nationally representative data set, so the finding is representative for decision-making purposes. This study was done using a zero-inflated Poisson regression model, which is best for analyzing a count response variable. So, this study differs from other studies in terms of the method of data analysis.

As limitations, mothers were asked to recall infants’ deaths in five years, so recall bias might exist. The cross-sectional nature of the study design might cause temporal relationships between dependent and independent variables. As long as some variables have high missed values and data were not collected for some variables in mini 2019 survey, important variables that determine infant death might not be included in this study.

## Abbreviations

AIC: Akaike information criterion
ANC: Antenatal care
BIC: Bayesian information criteria
CI: Confidence Interval
CSA: Central Statistical Agency
DHS: Demographic and Health Survey
EAs: Enumeration Areas
EDHS: Ethiopian Demographic and Health Survey
EMDHS: Ethiopia Mini Demography and Health Survey
EPHC: Ethiopian Population and Housing Census
EPHI: Ethiopian Public Health Institute
IRR: Incident rate ratio
IMR: Infant mortality rate
LLR: Log-likelihood Ratio
SNNPR: South nation nationality and people’s region
ZIP: Zero-inflated Poisson regression
